# Reliability and validity of the Polish version of the Achilles tendon Total Rupture Score

**DOI:** 10.1007/s00167-017-4764-7

**Published:** 2017-11-01

**Authors:** Paweł Bąkowski, Szymon Rubczak, Maria Wolff-Stefaniak, Monika Grygorowicz, Tomasz Piontek

**Affiliations:** 1 Orthopedic Department, Rehasport Clinic, Górecka 30, 60-201 Poznan, Poland; 2 Research and Development Department, Rehasport Clinic, Poznan, Poland; 3 Department of Spine Disorders and Pediatric Orthopedics, University of Medical Sciences Poznań, Poznan, Poland

**Keywords:** Achilles tendon rupture, ATRS, Cross-cultural, Validity, Reliability, Polish

## Abstract

**Purpose:**

The aim of this study was to perform the translation and cross-cultural adaptation of the Achilles tendon Total Rupture Score (ATRS) into Polish version, and to evaluate its reliability and validity.

**Methods:**

The ATRS was translated into Polish language according to the Beaton recommendations. A total number of 71 patients previously treated surgically (from 2011 to 2015), due to the Achilles tendon rupture, were enrolled in this study. ATRS-Polish was performed twice within a period of 5–10 days. To evaluate test–retest reliability, intra-rater coefficient (ICC) was calculated. Construct validity was determined by the Spearman’s rank coefficient correlation between the ATRS-Polish and a Polish version of EQ-5D-5L questionnaire.

**Results:**

Test–retest reliability was found to be excellent (ICC 0.9). The mean and standard deviation of the first and second assessment amounted 87.4 ± 14.0 and 88.4 ± 13.2, respectively. Construct validity analysis showed a strong correlation between the ATRS and the EQ-5D-5L score (*r* = − 0.69.) and moderate correlation between ATRS and actual comfort (*r* = 0.47).

**Conclusions and perspectives:**

Polish version of the Achilles tendon Total Rupture Score was found to be reliable and valid.

**Level of evidence:**

Level II.

**Electronic supplementary material:**

The online version of this article (doi:10.1007/s00167-017-4764-7) contains supplementary material, which is available to authorized users.

## Introduction

The Achilles tendon is the largest and strongest tendon in the human body [19]. Despite this fact, the Achilles tendon is considered the most commonly injured tendon in the lower extremity [1]. Current estimates show an incidence of about 5–10 ruptures (range 8.3–24) per 100,000 population [12, 13, 16, 22, 24]. The impact of Achilles tendon ruptures can be deeply felt across many aspects of patients’ lives. Therefore, an early diagnosis as well as choosing the proper treatment and rehabilitation programs is of crucial importance.

Up to date, the choice of Achilles tendon rupture treatment method is still ambiguous. Despite a vast array of reports, none of the published studies clearly demonstrate the definitive superiority of one method over another (as reviewed in [17]). In 2010, the American Academy of Orthopaedic Surgeons (AAOS) released 16 recommendations as clinical practice guidelines, based on a series of systematic reviews of published studies on the diagnosis and treatment of acute Achilles tendon rupture [7]. None of these was graded as strong recommendations which could be potentially used routinely [23]. Therefore, there is a need for creating an appropriate tool which aims at proper evaluation and reliable comparison of Achilles tendon ruptures treatment methods. Such evaluation of the treatment outcome, especially patient function, is of foreground importance, that is why patient-relevant instruments are commonly used.

The only patient-reported outcome measure (PROM) for Achilles tendon ruptures is the Achilles tendon Total Rupture Score (ATRS). It has been developed in 2007 in Sweden [19]. It consists of ten questions concerning current symptoms and physical activity during Achilles tendon rupture. As an outcome of this experimental setup, a one-factor score reflecting the desired dimension of symptoms and physical activity was achieved. This score comprised 10 items, 11-grade Likert scale each. Till now, ATRS has been successfully used in multiple patient groups, e.g. in the UK population with sustained and isolated acute Achilles tendon rupture [15]. Furthermore, recent studies demonstrated visible improvements in the ATRS during the first year following the surgery [4]. However, the successful employment of the ATRS strictly depends on the patients’ ability to read and to understand the language used in the questionnaire. Moreover, one has to take into account also the local culture of examined population. The incompatibilities of socio-cultural and economic conditions among different countries make the regular translation insufficient for proper estimation of the ATR score. For these reasons, the ATRS scale has been already translated and culturally adapted for English [5], Danish [9], Italian [26], Turkish [14], Portuguese [27], Dutch [9, 21] Persian [2] and recently also Chinese [8] and Norvegian [18]. Up to date, there is no Polish version of ATRS available, for that authors decided to translate and adapt this score into Polish.

The aim of this study was to perform the cross-cultural adaptation of the ATRS into Polish language and to evaluate its reliability and validity in a sample of Polish respondents.

## Materials and methods

### ATRS translation and cross-cultural adaptation

**Fig. 1:**
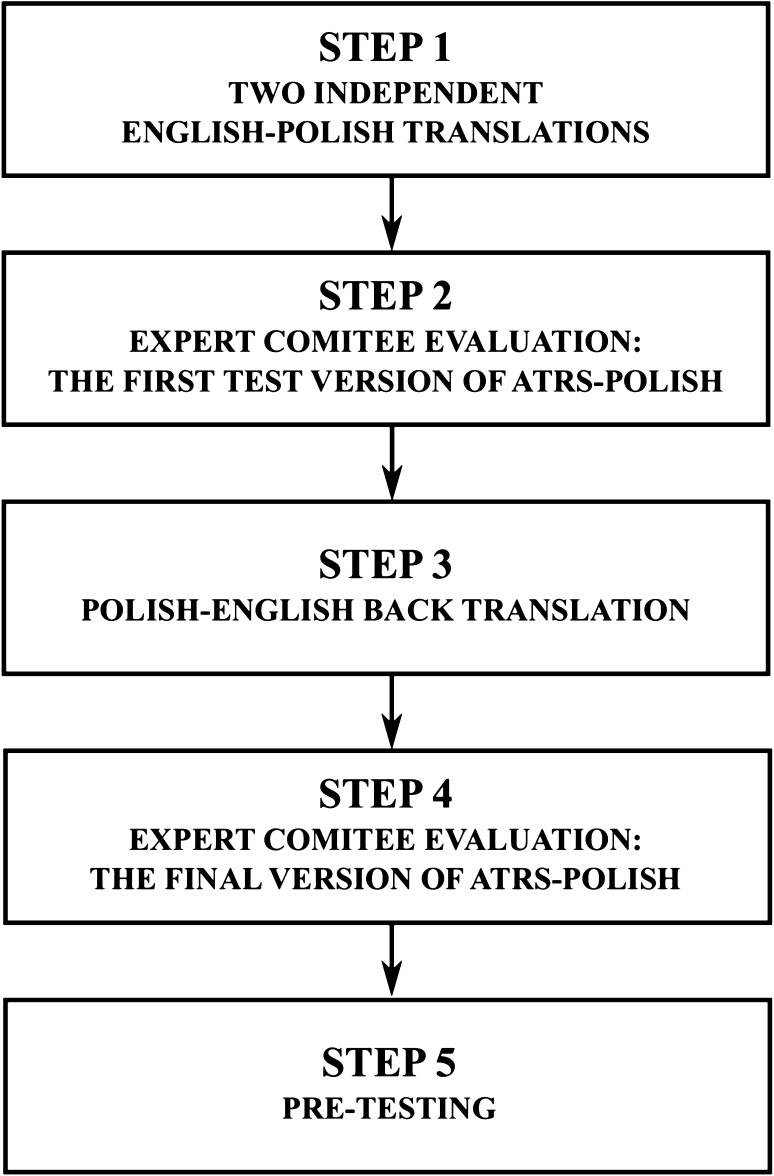
Flow chart of translation and cross-cultural adaptation of the Achilles tendon Total Rupture Score (ATRS) to Polish language

The ATRS was translated to Polish language according to the Beaton recommendations [3]. The whole translation procedure consisted of five following steps (Fig. 1):

*Step 1* Two native Polish speakers translated ATRS from English into Polish language. One translator was a non-medical, second was an orthopaedic surgeon. Both translations were performed independently. Only one translator had a prior knowledge of the questionnaire content.

*Step 2* Two distinct versions of the translated ATRS were presented to the expert committee consisting of four doctors and one physiotherapist with an expertise in Achilles tendon rupture treatment. The aim of such evaluation was to estimate the accuracy, fluency and style of translated scores as well as to discuss and highlight any possible errors or inconsistencies in translations. As an outcome, the first test version of Polish ATRS was established.

*Step 3* Back translation of the Polish ATRS to English language was performed by native English translator. The translator was unaware of the purpose of the present study and had no access to the original English version. This new translation into English allowed for identification of possible translation errors or grammatical inconsistencies, and was compared to the original version.

*Step 4* Expert committee reviewed and evaluated all versions of translated ATRS questionnaires. The aim of this step was to estimate the compatibility between all versions of test Polish ATRS translations. As an outcome, the final ATRS-Polish version was established and approved.

*Step 5* Pre-testing. ATRS-Polish was distributed among ten patients with ankle pain and among ten asymptomatic patients. None of the patients had difficulties in answering the questions. The feedback from the patients revealed the questions were clear and understandable.

### ATRS-polish validity

ATRS-polish questionnaire was compared to the Polish EQ-5D-5L test, previously adapted by Golicki et al. [10]. The EQ-5D-5L is a quality of life questionnaire which is widely used for clinical assessment. It consists of five questions regarding the mobility, self-care, usual activities, pain/discomfort and anxiety/depression. Each question comprises five levels (from 1 = no problem, to 5 = extreme problem). The less points EQ-5D-5L score gets, the better outcome it describes. Additionally, one question is related to the actual comfort (range from 0 to 100 points).

To assess convergent/divergent validity of ATRS-Polish, Spearman’s correlation coefficients and their 95% confidence intervals were calculated between ATRS-Polish and EQ-5D-5L as well as between ATRS-Polish and actual comfort. Spearman’s correlation coefficients were classified as follows: strong for values > 0.5; moderate for values between 0.35 and 0.5; weak correlation for values < 0.35 [11].

### ATRS-polish reliability

Test–retest reliability of ATRS-Polish was performed within a period of 5–10 days after performing the first test. No treatment was provided during that period to minimize the changes in patient’s clinical status. To assess consistency and agreement of test–retest reliability intraclass correlation coefficient (ICC) was used.

To evaluate internal consistency, which refers to homogeneity of all answers within a questionnaire, Cronbach coefficient alpha was used.

### Floor and ceiling effect

Floor and ceiling effect were also assessed, which refer to proportion of the respondents with the maximum (100 points) or minimum (0 points) scores. It is present when more than 30% of the respondents reach the maximum or minimum scores [14].

Statistical significance was accepted at *p* < 0.05. The calculations were performed using the PQStat 1.6.2 environment.

### Patients and ethics approval

A total number of 71 patients who presented Achilles tendon rupture and were treated in Poznań from 2011 to 2015 was enrolled in this study (Table 1). Patients were treated either in Rehasport Clinic in Poznań (49 cases) or in Poznań Regional Hospital (22 cases). 44 of them were treated with a percutaneous suturing, 17 patients by open suturing, in 1 case turn-down-flap suturing was performed and 9 patients received Achilles tendon reconstruction with semitendinosus and gracilis tendon grafts [20]. Left Achilles tendon was treated in 41 cases, in 30 cases right Achilles tendon was ruptured. A study was conducted 6–64 months after surgery (with a mean value of 30.0 +/- 13.0 months). 68 patients were eligible for inclusion in a reliability study. 3 patients were excluded from a validity study due to the missing data. The following inclusion criteria were applied: patient’s age over 18 years old and Polish nationality. Exclusion criteria were as follows: (i) age under 18 years old (ii) age over 60 years old and (iii) other lower limb injury. Invitation for participation in the study was performed during a phone call, where the goal as well as methodology of the study was presented. After that, patients were invited to the clinic, where after an examination by orthopaedic surgeon, the first test was performed. Patients received two questionnaires at the same time: ATRS-Polish and Polish version of EQ-5D-5L (previously adapted by Golicki [10]). The same test was performed after 5–10 days, then patients were asked during the telephone call to answer ATRS-Polish question one more time, to evaluate test–retest reliability. All patients gave written agreement for the participation in the study. This study was approved by the Bioethical Committee of the Regional Medical Council affiliated within Wielkopolska Izba Lekarska (opinion no. 191/2015, dated at 16.12.2015).

**Table 1:** Demographic and clinical characteristics of participants

Characteristics	Number or mean ± SD
Total number of patients	71
ATRS test participants	71
ATRS re-test participants	66
Age (year)	42.6 (± 8)
Gender	
Male (%)	71 (100%)
Female (%)	0 (0%)
Involved side	
Right (%)	30 (42%)
Left (%)	41 (58%)

### Sample size

There is no agreed optimum method for determining an appropriate sample size to evaluate aspects of validity for patient reported outcome measures [25]. Previous studies evaluating the aspects of validity and reliability of ATRS enrolled from 49 [5] to 112 patients [8]. We have reasoned that a case series of 71 patients would provide sufficient power to investigate the validity and reliability for the ATRS-Polish.

## Results

### Translating process

The procedure of translation and cultural adaptation of the original ATRS designed in Sweden into the Polish version was implemented (Fig. 1). The final ATRS-Polish is presented in Supplementary Fig. 1. The original questions were easily translated, in most cases the English words were directly translated into Polish ones. Therefore, the translation did not involve significant changes. Moreover, as already observed during pre-testing experiments which involved ten patients with ankle pain and ten asymptomatic ones, filling of the ATRS-Polish form did not rise any difficulties when answering the questions. All patients involved in pre-testing considered the ATRS-Polish form as easy to follow, user-friendly and well adapted to activities of daily living. This initial feedback from the patients clearly indicated that the questions were clear and understandable. The patients usually required 3–5 min to complete the form. The score values obtained in the first and second experiment are presented in Table 2.

**Table 2:** The absolute values of the ATRS-Polish and EQ-5D-5L scores

	Min	First quartile	Median	Mean	Third quartile	Max	SD	SE
ATRS test	28.0	81.0	91.0	87.4	97.0	100	14.0	1.6
ATRS re-test	29.0	84.5	92.0	88.4	99.0	100	13.2	1.6
Actual comfort	50.0	80.0	90.0	87.6	95.0	100	9.3	1.1
EQ-5D-5L	5	5	6	6	7	13	1.3	0.2

A mean value, median value, standard deviation (SD), standard error (SE), minimal and maximal values and first and third quartile of the outcomes are presented

### Validity

To validate the outcomes of newly constructed ATRS-Polish, the results were compared with already implemented EQ-5D-5L score in our clinic, previously adapted by Golicki et al. [10]. A strong correlation between the ATRS-Polish and a Polish EQ-5D-5L score was observed (*r* = − 0.69; *p* < 0.001, Table 3). Additionally, Polish-ATRS was moderately correlated with the actual comfort parameter (*r* = 0.47; *p* < 0.001).

**Table 3:** Validity as measured by correlation among questionnaires

Measurement	*r*	*p* value
ATRS vs EQ-5D-5L	− 0.691	< 0.000001
ATRS vs actual comfort	0.474	< 0.00001

Spearman’s correlation coefficients (*r*) for test–retest are presented

### Reliability

The test–retest reliability of the ATRS-Polish was found to be excellent with the ICC value of 0.9 (*p* < 0.0001). Internal consistency of the ATRS-Polish was found to be excellent (Cronbach’s alpha 0.93).

### Floor and ceiling effect

There were no missing data for individual items of ATRS since all patients responded to all questions (response rate 100%). The ATRS scores were well distributed and ranged between 28.0 and 100.0 (Table 2). No patient was scored the lowest score in test or re-test. Maximum score was observed in 15% of patients in the first assessment and in 20% of patients in the second assessment. No floor or ceiling effects were observed.

## Discussion

The most important finding of the present study was that the Polish version of the ATRS was reliable and valid and that the ATRS-Polish can be used in a Polish population to evaluate Achilles tendon rupture. Firstl, the translation of the original ATRS for a Polish context did not require major adaptations. All tested patients were able to correctly interpret and answer all activity-based questions included in the form. In this study, a combination of both, a paper-and-pencil questionnaire (during the first test) and a telephone survey (during the re-test) was applied. Such sampling appeared to be one of the strengths of the study, as almost 94% of patients enrolled in the first test decided to take part also in the second one. It could not be the case if a paper-and-pencil form for both tests (which would involve a second visit to the clinic) or an online survey (which could be highly dependent on the internet availability and/or the ability to proper use of the web tools) was enrolled. Moreover, a neutral setting of the responders during a telephone call in our study (in comparison to visit in a clinic which may influence the patients’ responses) additionally minimized the environmental biases.

To gain more insight into the reliability of the ATRS-Polish test, an extensive evaluation of the outcomes was performed. According to our results, the test was highly reproducible even when patients were asked to re-perform the test 10 days after initial study. The measured reliability was found to be excellent (ICC value of 0.90), which is in a good agreement with other studies based on translated versions of the original ATRS test: in Danish population (ICC = 0.91, [9]), Italian (ICC = 0.96, [26]), English (ICC = 0.98, [5]), Persian (ICC = 0.98, [2]) or Brazilian Portuguese (ICC = 0.93, [27]). By comparison, in the original ATRS study in Sweden [19], a significantly higher score was reported on the second test day compared with first test day when testing was performed twice within 2 weeks. The excellent test–re-test reliability indicated that the ATRS-Polish was stable over time and pointed into the possibility of detecting changes in patients with ATR. We can, therefore, reason that reproducibility of newly implemented ATRS-Polish test is definitely a strong point of the present study.

None of the patients achieved the minimum ATRS score, and therefore, no floor effect was observed. This is comparable to the results from other studies, which vary from 0 to 1% [9, 15]. 15% of patients achieved the maximum score in ATRS-test and 20% during the re-test, which is much more than the threshold observed in other studies (0–14%) [9, 14, 15]. The absence of floor or ceiling effects in the current study reflected the reliability, content validity, and responsiveness of the ATRS-Polish.

No missing items were observed and that the questions were clear and were of value were indicated to the patient, since questions in questionnaires were often marked as “not applicable” by the patient.

However, when individual questions were considered, particular patients did not respond equally, especially to question no. 7 in test and re-test. This question concerned quick walking up the stairs or hills. One of the possible explanations to that issue might be that not all of the patients were capable of such activities, independently of their Achilles tendon injury. This issue has been already discussed by Ganestam et al., when they proposed that questions no. 7, 8 (concerning running) and 9 (involves jumping) were not well adapted to all patients [9].

The construct validity of the ATRS-Polish questionnaire was determined by comparing the ATRS with selected outcome measures. For that purpose, a validated Polish version of EQ-5D-5L test has been used as well as the actual comfort score. The validity analysis showed strong correlation between the ATRS-Polish and EQ-5D-5L score (*r* = − 0.69). Additionally, it showed moderate correlation between ATRS and actual comfort (*r* = 0.47). These validation findings are in a good accordance with other studies concerning cross-national ATRS adaptations [2, 5, 9, 14, 18, 21, 26, 27]. It has been commonly accepted that such moderate strong correlation is desirable [6, 25], because quality of life EQ-5D-5L score is not specifically designed for Achilles tendon ruptures [10].

The major limitation of this study is that only men were enrolled in the study. It is, therefore, impossible to conclude about gender influence on the ATRS-Polish reliability and validity.

ATRS-Polish is easy to apply, reliable, valid, consistent and comparable to the original English version. It may, therefore, possess a significant clinical relevance. Validity and reliability, sufficient to assess clinical outcomes in the Polish population has been demonstrated. Significant benefits to both, practice and research, include the use of ATRS-Polish to identify patient’s symptoms and limitations and to monitor patient’s status over time. The therapeutic process can be enhanced by measuring the functional outcome and demonstrating the treatment effectiveness. Moreover, validated and reliable Polish version of an international ATRS test will definitely allow Polish clinicians to compare the results of their patients on a nationwide scale.

## Conclusions

Summarizing, we propose for the first time a Polish version of the ATRS test. The possible use of the cross-culturally adapted ATRS-Polish may be considered to evaluate the clinical condition after Achilles tendon rupture in day-by-day clinical practice in the Polish population. The implementation of ATRS-Polish test will enhance the whole therapeutic process of Achilles tendon rupture.

## Electronic supplementary material

Below is the link to the electronic supplementary material.

Supplementary material 1 (PDF 269 KB)
