# Supplementary material for: Reliability and validity of the Polish version of the Achilles tendon Total Rupture Score
Source: Knee Surg Sports Traumatol Arthrosc. 2017 Nov 1;26(7):2074–9. doi: 10.1007/s00167-017-4764-7 (PMC6061432; doi:10.1007/s00167-017-4764-7)
Supplement: Supplementary file 1 — Supplementary material 1 (PDF 269 KB) [file 167_2017_4764_MOESM1_ESM.pdf]

Skala oceny po zerwaniu ścięgna Achillesa

Zakreślamy na poziomej linii w skali 0-10.
0- bez ograniczeń, 10- poważne ograniczenia

1. Czy masz jakieś ograniczenia związane z obniżeniem siły mięśni łydki/ścięgna Achillesa/stopy?

   0 1 2 3 4 5 6 7 8 9 10

2. Czy masz jakieś ograniczenia związane z męczliwością mięśni łydki/ścięgna Achillesa/stopy?

   0 1 2 3 4 5 6 7 8 9 10

3. Czy masz jakieś ograniczenia związane ze sztywnością mięśni łydki/ścięgna Achillesa/stopy?

   0 1 2 3 4 5 6 7 8 9 10

4. Czy masz jakieś ograniczenia związane z bólem mięśni łydki/ścięgna Achillesa/stopy?

   0 1 2 3 4 5 6 7 8 9 10

5. Czy masz jakieś ograniczenia w codziennej aktywności?

   0 1 2 3 4 5 6 7 8 9 10

6. Czy masz jakieś ograniczenia podczas chodzenia na nierównym podłożu?

   0 1 2 3 4 5 6 7 8 9 10

7. Czy masz jakieś ograniczenia podczas szybkiego wchodzenia po schodach/chodzeniu pod górę?

0 1 2 3 4 5 6 7 8 9 10

8. Czy masz jakieś ograniczenia w bieganiu?

0 1 2 3 4 5 6 7 8 9 10

9. Czy masz jakieś ograniczenia w skakaniu?

0 1 2 3 4 5 6 7 8 9 10

10. Czy masz jakieś ograniczenia w wykonywaniu ciężkiej pracy fizycznej?

0 1 2 3 4 5 6 7 8 9 10
